# Dominantly acting *ABCC8* mutations in patients with medically unresponsive hyperinsulinaemic hypoglycaemia

**DOI:** 10.1111/j.1399-0004.2010.01476.x

**Published:** 2011-06

**Authors:** SE Flanagan, RR Kapoor, I Banerjee, C Hall, VV Smith, K Hussain, S Ellard

**Affiliations:** 1 Institute of Biomedical and Clinical Science, Peninsula Medical School, University of ExeterExeter, UK; 2 >London Centre for Paediatric Endocrinology and Metabolism, Great Ormond Street Hospital for Children NHS TrustLondon, UK; 3 >The Institute of Child Health, University College LondonLondon, UK; 4 >Northern Congenital Hyperinsulinism (NORCHI) Service, Royal Manchester Children's HospitalManchester, UK

**Keywords:** *ABCC8*, diazoxide, hyperinsulinaemic hypoglycaemia, pancreatectomy

## Abstract

Flanagan SE, Kapoor RR, Banerjee I, Hall C, Smith VV, Hussain K, Ellard S. Dominantly acting *ABCC8* mutations in patients with medically unresponsive hyperinsulinaemic hypoglycaemia.

Recessive inactivating mutations in the *ABCC8* and *KCNJ11* genes encoding the adenosine triphosphate-sensitive potassium (K_ATP_) channel subunit sulphonylurea receptor 1 (SUR1) and inwardly rectifying potassium channel subunit (Kir6.2) are the most common cause of hyperinsulinaemic hypoglycaemia (HH). Most of these patients do not respond to treatment with the K_ATP_ channel agonist diazoxide. Dominant inactivating *ABCC8* and *KCNJ11* mutations are less frequent, but are usually associated with a milder form of hypoglycaemia that is responsive to diazoxide therapy. We studied five patients from four families with HH who were unresponsive to diazoxide and required a near total pancreatectomy. Mutations in *KCNJ11* and *ABCC8* were sought by sequencing and dosage analysis. Three novel heterozygous *ABCC8* mis-sense mutations (G1485E, D1506E and M1514K) were identified in four probands. All the mutations affect residues located within the Nucleotide Binding Domain 2 of the SUR1 subunit. Testing of family members showed that the mutations had arisen *de novo* with dominant inheritance in one pedigree. This study extends the clinical phenotype associated with dominant K_ATP_ channel mutations to include severe congenital HH requiring near total pancreatectomy in addition to a milder form of diazoxide responsive hypoglycaemia. The identification of dominant *vs* recessive mutations does not predict clinical course but it is important for estimating the risk of HH in future siblings and offspring.

The pancreatic *β*-cell adenosine triphosphate-sensitive potassium (K_ATP_) channel is a complex of four sulphonylurea receptor 1 (SUR1) with four inwardly rectifying potassium channel subunits (Kir6.2) that play a pivotal role in regulating insulin secretion (1). Metabolic regulation of K_ATP_ channel activity is mediated by changes in the intracellular concentrations of adenosine triphosphate (ATP) and MgADP, which inhibit or activate the channel respectively (1, 2). Sulphonylureas and diazoxide bind to the SUR1 subunits to, respectively, close or open the channels independently of ATP/adenosine diphosphate (ADP) concentrations and stimulate or inhibit insulin secretion.

Hyperinsulinaemic hypoglycaemia (HH) is characterized by the dysregulation of insulin secretion that often presents in the neonatal period. Early diagnosis is important to avoid irreversible brain damage because of prolonged hypoglycaemia (3). The clinical presentation is heterogeneous ranging from mild to severe symptoms of hypoglycaemia. Inactivating mutations in the genes *ABCC8* and *KCNJ11* encoding the SUR1 and Kir6.2 subunits of the K_ATP_ channel are the most common cause of HH (4, 5). Histologically, there are diffuse and focal forms of the disease. Focal lesions arise from paternal uniparental isodisomy of the chromosome 11p15 region within the embryonic pancreas of a foetus with a paternally inherited K_ATP_ channel mutation (6). Surgical removal of the lesion is usually curative. A similar number of cases have recessively inherited the loss of function mutations where the entire pancreas is affected (diffuse). This usually leads to severe, medically unresponsive HH often requiring a near total pancreatectomy. The lack of response to diazoxide is believed to be because of the absence of the K_ATP_ channels at the membrane (if the mutation results in lack of protein product or affects maturation/transport to the membrane) or the presence of inactive K_ATP_ channels that cannot respond to diazoxide.

Dominant inactivating mis-sense mutations in *ABCC8* and *KCNJ11* are a rare cause of HH (7, 8). To date the phenotype of patients with heterozygous mutations seems to be different to those with recessive mutations as they show responsiveness to diazoxide. This response may be attributed to the binding of diazoxide to a normal SUR1 subunit within the heteromeric complex or to the presence of a sufficient proportion of normal K_ATP_ channels as a consequence of the binomial distribution (9). Huopio et al. described the first dominantly inherited *ABCC8* mutation, E1507K (described by Huopio et al. as E1506K based on isoform L78207 that excludes the alternatively spliced amino acid in exon 17), that caused HH in early life and predisposes to later insulin deficiency. All the patients described in this large family had a mild form of HH that could be managed by long-term diazoxide treatment (7). Recently, Pinney et al. have reported 14 different dominantly inherited *ABCC8* or *KCNJ11* mis-sense mutations in 16 families with HH that often escaped detection in infancy and responded to diazoxide (8). We now describe five patients with heterozygous *ABCC8* mutations who presented with severe, medically unresponsive HH and underwent near total pancreatectomy.

## Materials and methods

### Subjects

We studied five patients from four families with HH. In one family, a mother and a son were affected, whereas for the three remaining probands there was no family history of HH. As a result of the failure to respond to medical therapy, all patients underwent a near total pancreatectomy. This study was conducted in accordance with the Declaration of Helsinki and informed consent was obtained from all patients, with parental consent given on behalf of children.

### Molecular genetics

Genomic DNA was extracted from peripheral leukocytes and pancreatic tissue using standard procedures and the single exon of the *KCNJ11* gene was sequenced as previously described (10). When no *KCNJ11* mutation was identified, the 39 exons of *ABCC8* were amplified and sequenced (11). Sequences were compared to the published sequence, NM_000352.2, that incorporates the alternatively spliced residue in exon 17 (L78208, L78224). Mutation testing was performed on parental DNA extracted from peripheral leukocytes. When a *de novo* mutation was identified, microsatellite analysis confirmed family relationships. In one family as the unaffected maternal grandfather was deceased, haplotype analysis was performed using six microsatellite markers on chromosome 11p15 to determine the origin of the mutation. Novel mutations were tested by sequencing 500 control chromosomes and dosage analysis of the *ABCC8* gene was undertaken for all probands (MRC Holland, Amsterdam, the Netherlands, multiplex ligation-dependent probe amplification kit P117). Pancreatic tissue was available from two probands and loss of heterozygosity was investigated by microsatellite analysis of chromosome 11p15 (12).

### Histology/immunohistochemistry

Paraffin sections (3 µm thick) were cut from blocks of resected pancreatic tissue from the four probands and were stained with haematoxylin and eosin (H & E). Immunostaining was performed using polyclonal antibodies for insulin, somatostatin, glucagon and pancreatic polypeptide as previously described (13). Pancreatic tissue was not available from the mother of proband 2 who underwent a pancreatectomy 23 years ago.

## Results

### Molecular genetics

**Fig 1:**
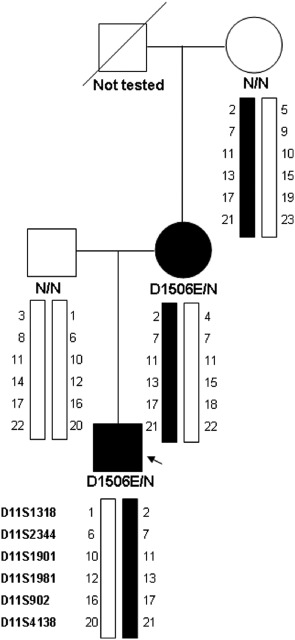
Microsatellite analysis of markers spanning 15.5 Mb on chromosome 11p15.5-15.1 in family 2 with hyperinsulinaemic hypoglycaemia (HH) resulting from a heterozygous D1506E mutation in the *ABCC8* gene. *ABCC8* is located between markers D11S1981 and D11S902. *Black bars* indicate the haplotype co-segregating with hyperinsulinism. *Squares* denote males, *circles* females, and *black symbols* show individuals affected with HH. Genotypes are provided below each symbol and an arrow points to the proband.

Sequencing of the *KCNJ11* gene failed to detect any mutations but subsequent analysis of the *ABCC8* gene identified three different novel heterozygous mis-sense mutations in the five patients (Table 1). The D1506E (c.4518C>A) mutation was seen in three patients from two families. In family 1, testing of the unaffected parents showed that the mutation had arisen *de novo* in the proband. In family 2, the mutation had been inherited from the affected mother. Testing was undertaken for the unaffected maternal grandmother who did not carry the D1506E mutation. No DNA was available from the deceased unaffected grandfather, but haplotype analysis of chromosome 11p15 markers suggested that the D1506E mutation had arisen *de novo* on the grandmaternal chromosome (Fig. 1). The G1485E (c.4454G>A) and M1544K (c.4541T>A) mutations were each identified in one patient. Testing of their unaffected parents showed that the mutations had arisen *de novo*. All three mutations affect highly conserved residues within the Nucleotide Binding Domain 2 (NBD2) of SUR1, were not present in 500 normal control chromosomes and a second *ABCC8* mutation was not detected by dosage analysis. Analysis of markers on chromosome 11p15 in DNA extracted from the resected pancreatic tissue of probands from families 1 and 4 showed biparental inheritance with no evidence for loss of heterozygosity.

**Table 1:** Summary of the clinical features, treatment, genetic and histological findings in patients with dominant *ABCC8* mutations

Family	Birth weight (g)	Gestation (weeks)	Age at presentation	Maximum glucose requirement (mg/kg/min)	Diazoxide responsive (20 mg/kg/day)	Age at surgery (subtotal pancreatectomy; weeks)	Histology	11p15.5-11p15.1 microsatellite analysis of pancreatic tissue	Post-operative management	Mutation	Inheritance
1	4640	39	Day 1	Not known	No	4 and 12	Diffuse disease	Heterozygous	Octreotide and increased feeds for recurrence of hypoglycaemia	D1506E	*De novo*
2	3190	33	Day 1	18	No	8	Diffuse disease	Not known	Insulin (diabetes diagnosed post-operatively)	D1506E	Inherited from affected mother
	4528	39	Day 3	16	No	5	Not known	Not known	Diazoxide and/or octreotide with increased feeds till 11 years. Insulin at 23 years during pregnancy	D1506E	*De novo*
3	3900	40	Day 1	17	No	8	Diffuse disease	Not known	Insulin (diabetes diagnosed post-operatively)	M1514K	*De novo*
4	3500	36	Day 1	15	No	8	Diffuse disease	Heterozygous	Octreotide, diazoxide and increased feeds	G1485E	*De novo*

### Histology/immunohistochemistry

**Fig 2:**
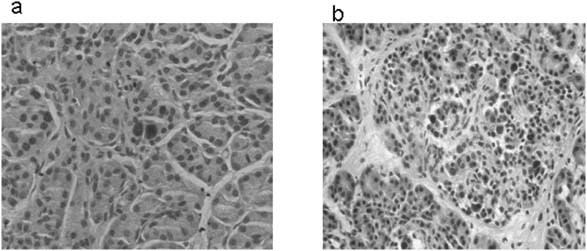
(a and b) Comparison of the histology of the resected pancreas from the proband of family 2 and a patient with recessively inherited congenital hyperinsulinism because of *ABCC8* mutations. (a) Haematoxylin & eosin (H & E)-stained sections showing the presence of scattered endocrine cells with large and frequently giant endocrine nuclei. The endocrine tissue was poorly organized into islets. (b) H & E-stained sections of pancreatic tissue from a patient with recessive *ABCC8* mutations showing the large and frequently giant endocrine nuclei and irregularly sized islets.

Histological analysis of pancreatic material from the four probands with dominant *ABCC8* mutations showed large giant endocrine nuclei in scattered cells that stained positive for insulin antibodies, which is not dissimilar to the changes that are observed in patients with recessive *ABCC8* mutations (Table 1 and Fig. 2). Pancreatic polypeptide-positive cells were also observed and immunostaining for somatostatin and glucagon was unremarkable.

### Clinical characteristics

All five patients presented with severe HH immediately after birth and four patients were macrosomic (birth weight >90th percentile; Table 1). None responded to maximum doses of diazoxide (20 mg/kg/day) and normoglycaemia could not be maintained despite using high-calorie feeds and octreotide. The probands from families 2 and 3 had a near total pancreatectomy at 8 weeks of age and subsequently developed diabetes mellitus. Proband 4 and the mother of proband 2 underwent a near total pancreatectomy at 8 and 5 weeks of age, respectively, but post-operatively they continued to have HH that was managed on a combination of subcutaneous octreotide injections and frequent high-calorie carbohydrates feeds. Although proband 4 is still on treatment at the age of 3, the mother of proband 2 outgrew the HH at 11 years of age, but developed impaired glucose tolerance at 14 years of age and required insulin at 23 years of age during her third trimester of pregnancy. Proband 1 underwent an initial near total pancreatectomy but severe post-operative hypoglycaemia required a second total pancreatectomy. Despite this, the patient still requires continuous high-calorie feeds and octreotide to maintain normoglycaemia (Table 1).

## Discussion

We identified three novel heterozygous *ABCC8* mis-sense mutations (G1485E, D1506E and M1514K) in five patients from four families. The mutations had arisen *de novo* in all four pedigrees. Although a second mutation was not found in any of the five patients by sequence or dosage analysis in leukocyte DNA, the presence of a mutation in the regulatory regions of the gene or as a result of a somatic event during pancreatic development cannot be excluded. However, as histological examination and chromosome 11p15 microsatellite marker analysis of resected pancreas, in four and two patients respectively, were consistent with diffuse disease, we conclude that G1485E, D1506E and M1514K are dominantly acting *ABCC8* mutations associated with diffuse disease and a 50% risk of HH in future offspring.

All the five patients presented with severe HH within the first 3 days of life with no response to maximum doses of diazoxide (20 mg/kg/day) and subsequently underwent near total pancreatectomy. This clinical presentation is very different to that described in previous reports of dominant *ABCC8* mis-sense mutations that were associated with a milder phenotype (7, 8). The mutations identified in our series are novel, suggesting a genotype/phenotype correlation where different mis-sense mutations may result in a variable severity of HH.

Pancreatic *β*-cell K_ATP_ channels are regulated by intracellular nucleotides (ATP and ADP). SUR1 has two cytoplasmic nucleotide-binding domains (NBD1 and NBD2) that sense changes in intracellular [ATP]/[ADP] and transmit the signal to the pore. Binding of MgATP to NBD2 leads to its hydrolysis to MgADP, which then activates the K_ATP_ channel (14). The ability of diazoxide to activate the channel depends on the simultaneous presence of MgATP and/or MgADP and functional NBDs (15–17). Mutations in NBD2 can therefore abolish channel activation by diazoxide or MgADP (15). This loss of activation by MgADP on K_ATP_ channel activity when metabolism is reduced has been reported for patients with HH because of recessive *ABCC8* mutations and seems to be a common molecular mechanism of HH (18, 19).

Nine of the 11 previously reported dominant *ABCC8* mutations affect residues within the NBD2 region and functional studies have shown that these mutations significantly diminish or completely abolish the channels' response to MgADP and diazoxide (7, 8). Further studies on 10 of the *ABCC8* mutations, under simulated heterozygous conditions, indicated that although the mutant subunits do reduce channel function, the wild-type *ABCC8* allele is sufficient to confer partial channel response to changes in the ATP/ADP ratio expected during glucose metabolism and to diazoxide (8). The three mutations identified in this series of patients are located in the NBD2 region of SUR1 and it is likely that they also abolish the stimulatory effects of MgADP. This hypothesis is supported by the identification of the same mutation, D1506E, in three individuals from two families with diazoxide-unresponsive HH.

Our findings suggest that the clinical presentation of patients with dominant inactivating *ABCC8* mis-sense mutations is variable, ranging from mild medically responsive to severe early onset HH requiring a near total pancreatectomy. The clinical presentation of patients at the time of diagnosis cannot distinguish between recessive and dominantly acting *ABCC8* mutations. A genetic diagnosis is important because finding a *de novo* dominant mutation in a sporadic case with diazoxide-unresponsive HH confers a lower recurrence risk for future siblings compared to a recessive mutation, but a higher risk of HH (50%) for the next generation.
